# Involvement of JNK signaling in *Aspergillus fumigatus*-induced inflammatory factors release in bronchial epithelial cells

**DOI:** 10.1038/s41598-023-28567-3

**Published:** 2023-01-23

**Authors:** Xiao Cui, Fangyan Chen, Jingya Zhao, Dingchen Li, Mandong Hu, Xue Chen, Yulin Zhang, Li Han

**Affiliations:** 1 Department of Respiratory and Critical Care Medicine, Beijing Youan Hospital, Capital Medical University, Beijing, 100069 China; 2 Department for Disinfection and Infection Control, Chinese PLA Center for Disease Control and Prevention, Beijing, China; 3 National Center of Biomedical Analysis, 27 Taiping Lu, Beijing, 100850 China

**Keywords:** Immunology, Microbiology

## Abstract

*Aspergillus fumigatus* (*A. fumigatus*) is an important fungal pathogen and its conidia can be inhaled and interact with airway epithelial cells; however, the release of inflammatory factors from bronchial epithelial cells upon *A. fumigatus* infection and its regulation remained unclear. Here it was demonstrated that the release of IL-27, MCP-1 and TNF-α from BEAS-2B cells were upregulated upon stimulation by conidia, while mitogen-activated protein kinase signaling pathway was activated. Further, the inhibition of JNK, but not p38 and ERK, could inhibit inflammatory factors release and the LC3II formation in BEAS-2B cells induced by *A. fumigatus* conidia. In addition, an inhibitor of autophagy, bafilomycin A1 was able to significantly down-regulate the release of inflammatory factors in BEAS-2B cells upon *A. fumigatus* conidia, while rapamycin could reverse the effect of JNK inhibitor on IL-27 and TNF-α release. Taken together, these data demonstrated that JNK signal might play an important role in inflammatory factor release regulated by autophagy in bronchial epithelial cells against *A. fumigatus* infection.

## Introduction

Invasive pulmonary aspergillosis (IPA) induced by *Aspergillus fumigatus* (*A. fumigatus*) becomes a major health problem due to the increasing number of susceptible populations^1^. The conidia of *A. fumigatus* could be inhaled and deeply embedded into the human airway^2^. Previous studies on the pathogenesis of IPA usually focused on the interaction of *A. fumigatus* with professional immune cells, such as macrophages, neutrophils^3^. However, accumulating evidence has implicated that airway epithelial cells, including bronchial epithelial cells and lung epithelial cells, also play important roles in the host innate immunity against *A. fumigatus*^4^*.*

The production of cytokines has been proved to play a critical role in fungal clearance^5^. In alveolar macrophages, the activation of MyD88/NF-κB and Syk/PI3K was suggested to participate in *A. fumigatus*-induced inflammatory response^6^. Moreover, autophagy has been shown to closely intertwined with inflammatory response and proinflammatory cytokine secretion in macrophages^7,8^. In lower respiratory tract, the inhaled *A. fumigatus* conidia interact with airway epithelial cells and initiate the activation of subsequent intracellular signaling pathways and the release of cytokines and chemokines^9,10^. However, due to the complexity of cell types and different morphotypes of conidia (resting conidia, swollen germinating conidia or hyphae), the regulation on the release of inflammatory factors in the airway epithelial cells induced by *A. fumigatus* are not sufficiently comprehended^11^.

Previous studies indicated that early inflammatory responses protect the host, while hyperinflammatory state impaired innate immune functions^12^. Dysregulated proinflammatory signaling cascade need negative-feedback mechanisms to constrain the excessive inflammatory response. Mitogen-activated protein kinase (MAPK) signaling pathway played an important role in autophagy regulation and the production of proinflammatory cytokines during pathogen infection, such as *Mycobacterium tuberculosis*, Influenza virus and *Streptococcus pneumoniae*^13–16^. MAPKs are activated by specific upstream kinases through reversible phosphorylation of both threonine and tyrosine residues of the TXY motif. Conversely, the dephosphorylation of either residue is sufficient for inactivation of MAPKs, which could be achieved largely by dual-specificity protein phosphatases (DUSPs) specific for MAPKs^17^. More than 11 different DUSPs have been identified that are highly specific for MAPKs but differ in substrate specificity to MAPKs^18^.

In this study the production of cytokines induced by *A. fumigatus* conidia in human bronchial epithelial cells BEAS-2B were investigated and the potential involvement of autophagy and MAPK pathways in this process were also evaluated.

## Results

### Release of inflammatory factors from bronchial epithelial cells stimulated by *A. fumigatus* conidia

**Figure 1:**
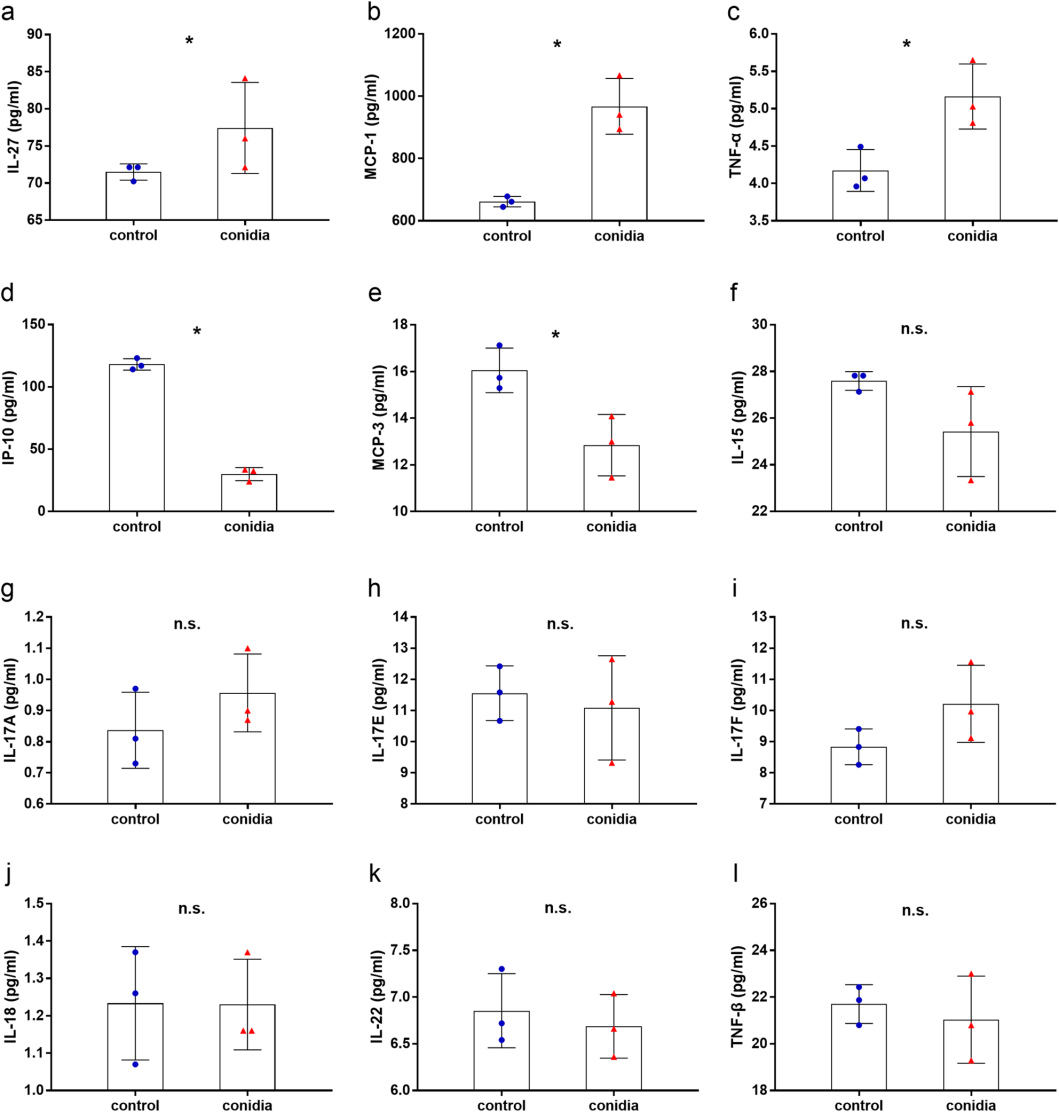
Release of proinflammatory cytokines from bronchial epithelial cells stimulated by *A. fumigatus* conidia. BEAS-2B cells were stimulated with resting conidia of *A. fumigatus* conidia at an MOI of 10 for 8 h. The secretion of IL-27, MCP-1, TNF-α, IP-10, MCP-3, IL-15, IL-17A, IL-17E, IL-17F, IL-18, IL-22 and TNF-β in supernatant of each group was detected (**a**–**l**). Data are presented as mean ± SD (n = 3). *P < 0.05; *n.s*. not significant; Student's *t* test.

It was reported that inflammatory factors released by macrophages were involved in antifungal immune response to *A. fumigatus* infection^19^. Here, the release of cytokines in bronchial epithelial cells was examined at 8 h post infection by *A. fumigatus* conidia with the multiplicity of infection (MOI) of 10. It was found that upon stimulation by conidia more interleukin (IL)-27, monocyte chemoattractant protein (MCP)-1, tumor necrosis factor (TNF)-α were released by BEAS-2B cells, whereas less IFN-γ-inducible protein (IP)-10 and MCP-3 were presented compared to the control group (*P* < 0.05) (Fig. 1a–e). Moreover, the secretion of IL-15, IL-17A, IL-17E, IL-17F, IL-18, IL-22 and TNF-β remained unchanged in conidia stimulation group compared with control group (*P* > 0.05) (Fig. 1f–l). These results showed that the release of inflammatory factors from BEAS-2B cells were differently regulated during the infection of *A. fumigatus*.

### Activation of MAPK signaling pathways by *A. fumigatus* conidia in bronchial epithelial cells

**Figure 2:**
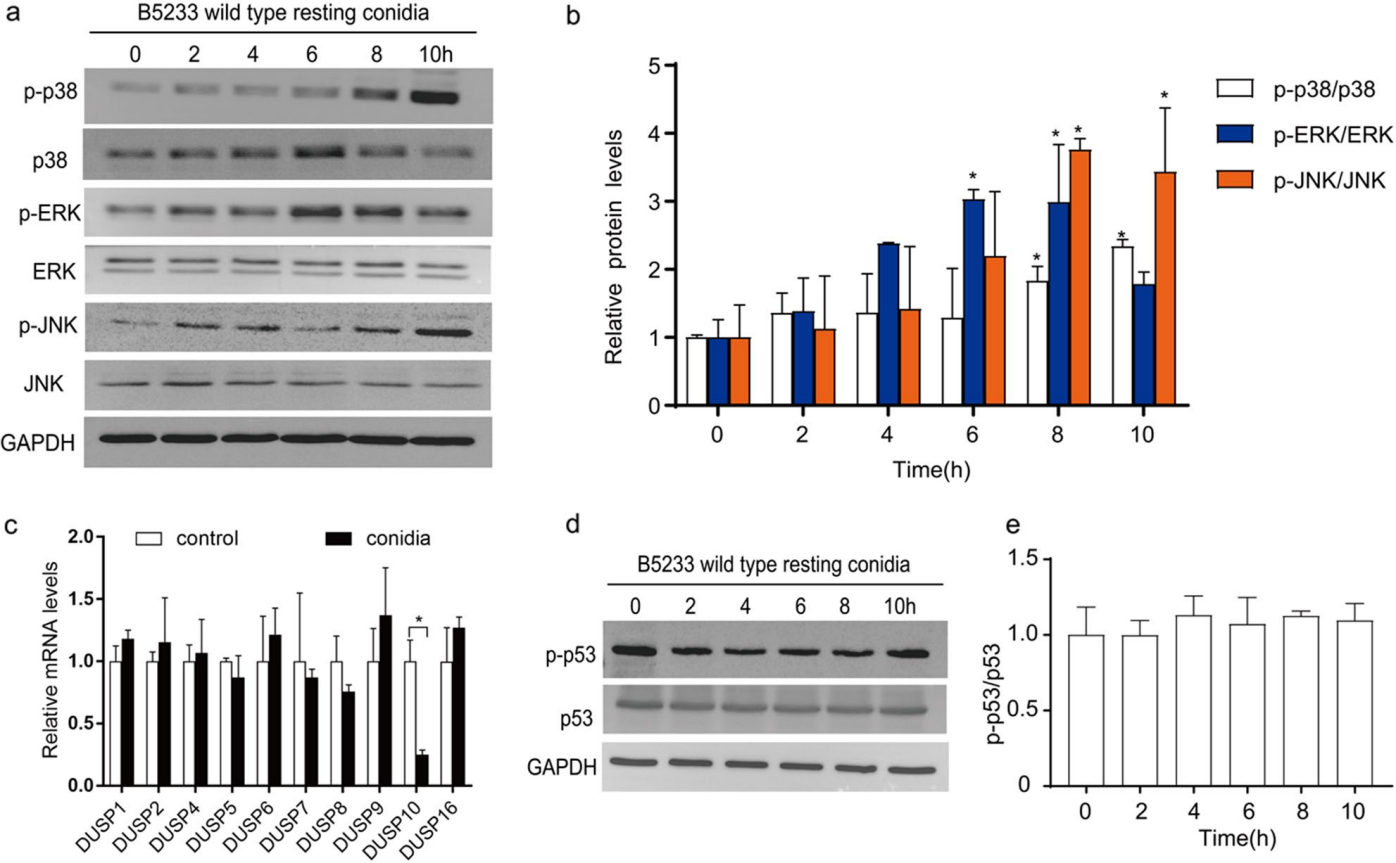
Activation of MAPK signaling pathways by *A. fumigatus* conidia in bronchial epithelial cells. BEAS-2B cells were stimulated with resting conidia of *A. fumigatus* at an MOI of 10 for the indicated times. (**a**,**b**) Phosphorylation of p38 MAPK, ERK and JNK were measured and analyzed. The blots were cut prior to hybridisation with antibodies during blotting. (**c**) The mRNA level of DUSPs was measured by RT-qPCR after stimulated for 8 h. (**d**,**e**) Phosphorylation of p53 were measured and analyzed. The blots were cut prior to hybridisation with antibodies during blotting. The data shown as mean ± SD and represented 3 independent experiments. *P < 0.05 versus the control group.

Since it is known that MAPK signaling pathways are the key upstream signals in inducing inflammatory factors production^20^, we explored the possible involvement of MAPK pathways in the regulation of *A. fumigatus*-induced cytokines production in BEAS-2B cells*.* First, the expression and phosphorylation of three major kinases, p38 MAPK, ERK and JNK in MAPK signaling pathway were determined in BEAS-2B cells upon stimulation by *A. fumigatus* conidia. Western blotting showed that the phosphorylation levels of p38 MAPK, ERK and JNK significantly increased with stimulation by *A. fumigatus* conidia (Fig. 2a,b).

To further confirm the upregulation of MAPK pathways by conidia, the expression of inhibitory factors specific for MAPKs was also investigated. DUSPs are a group of protein phosphatases that can dephosphorylate and inactivate MAPKs. As shown in Fig. 2c, only the mRNA level of DUSP10 was downregulated, whereas most DUSPs were not affected after exposure to *A. fumigatus* conidia in BEAS-2B cells. These data gave evidence from the opposite side that *A. fumigatus* could activate the MAPK signaling pathways in bronchial epithelial cells. Further, since it was known that p53 protein can functionally interact with MAPK, leading to p53-mediated cellular responses^21^, the phosphorylation level of p53 in BEAS-2B cells was further examined. As shown in Fig. 2d,e, both expression and phosphorylation level of p53 were not affected by *A. fumigatus* conidia. Taken together, MAPK pathways in bronchial epithelial cells were activated by *A. fumigatus* conidia without affecting p53. Thereby, the signaling regulating the release of inflammatory factors need further investigation.

### JNK signal regulated the release of cytokines induced by *A. fumigatus* in bronchial epithelial cells

**Figure 3:**
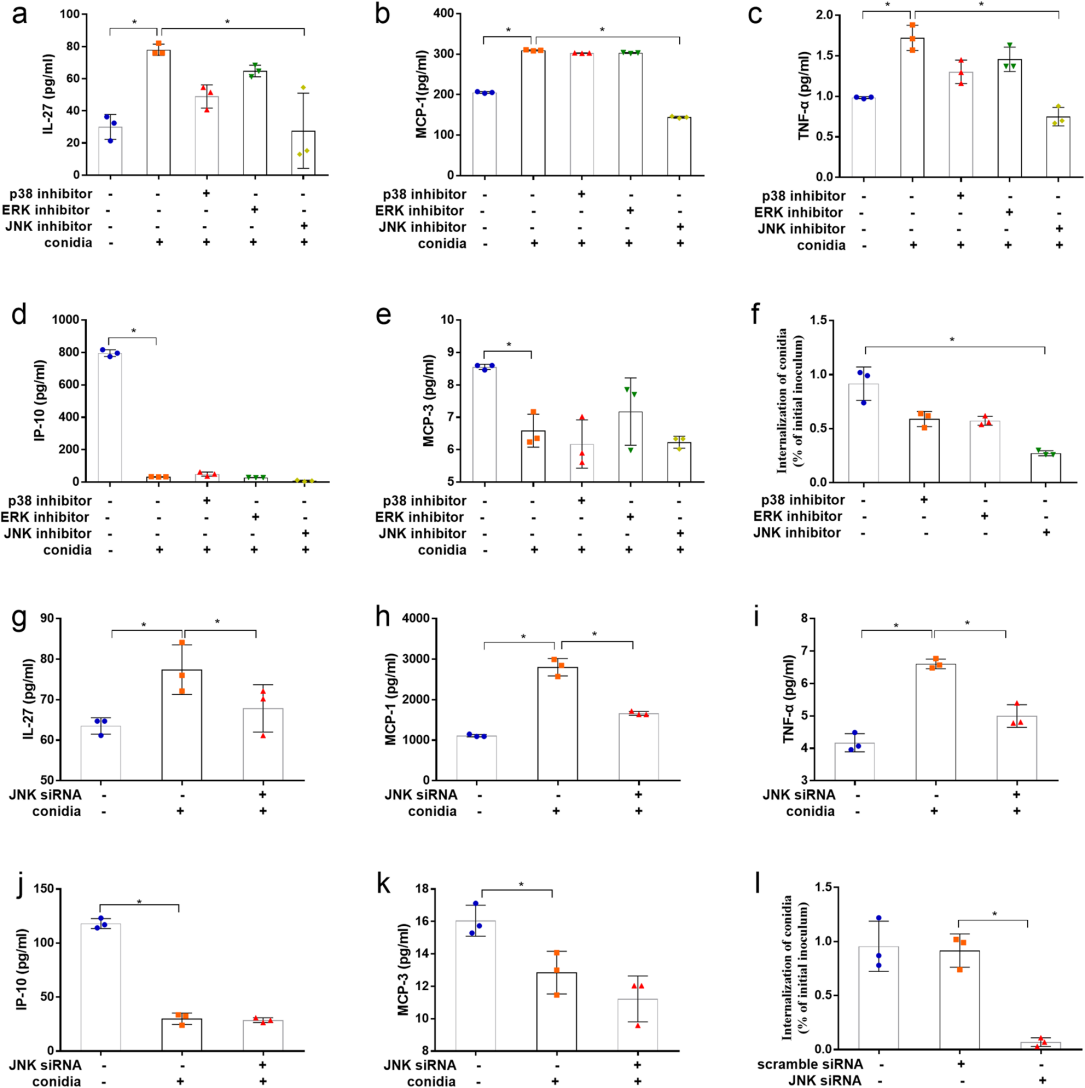
Involvement of JNK signaling pathway in internalization of *A. fumigatus* and release of cytokines. Prior to inoculation with *A. fumigatus* conidia for 8 h, BEAS-2B cells were treated with 20 μmol p38 inhibitor SB203580, 20 μmol ERK inhibitor U0126, 20 μmol JNK inhibitor SP600125 for 1 h (**a**–**f**), or transfected with siRNA targeting JNK for 36 h (**g**–**l**). The secretion of IL-27, TNF-α, MCP-1, IP-10 and MCP-3 in supernatant of each group was detected (**a**–**e**,**g**–**k**). Internalization of conidia was assessed by the nystatin protection method (**f**,**l**). Data are presented as mean ± SD (n = 3, *P < 0.05).

Further, we testified whether MAPK pathways contribute to the release of inflammatory cytokines in bronchial epithelial cells induced by *A. fumigatus* conidia. First, three inhibitors of MAPKs, SB203580 for p38 MAPK, U0126 for ERK and SP600125 for JNK were used in our experiment respectively. As shown in Fig. 3a–e, neither p38 nor ERK inhibitors affected the release of inflammatory cytokines, whereas JNK inhibitors effectively blocked the induction of IL-27, MCP-1 and TNF-α by conidia (*P* < 0.05). All the three inhibitors did not affect the inhibitory effect of conidia on IP-10 and MCP-3 (*P* > 0.05). Moreover, pretreatment by JNK inhibitor of BEAS-2B cells significantly inhibited the internalization of *A. fumigatus* conidia. However, p38 or ERK inhibitors had no effect on internalization of conidia (Fig. 3f).

Similarly, transfection of siRNA for JNK in BEAS-2B cells reduced the release of IL-27, MCP-1 and TNF-α induced by *A. fumigatus* (*P* < 0.05) (Fig. 3g–i). Nevertheless, the expression of IP-10 and MCP-3 remained unchanged in JNK siRNA transfection group when compared to conidia stimulation group (*P* > 0.05) (Fig. 3j,k). Moreover, JNK siRNA also inhibited the internalization of *A. fumigatus* conidia into bronchial epithelial cells (Fig. 3l). These results indicated that JNK, but not p38 MAPK and ERK, might play an important role on the interaction between *A. fumigatus* and bronchial epithelial cells, including internalization of *A. fumigatus* and release of inflammatory factors.

### Involvement of JNK in* A. fumigatus* conidia-induced autophagy in bronchial epithelial cells

**Figure 4:**
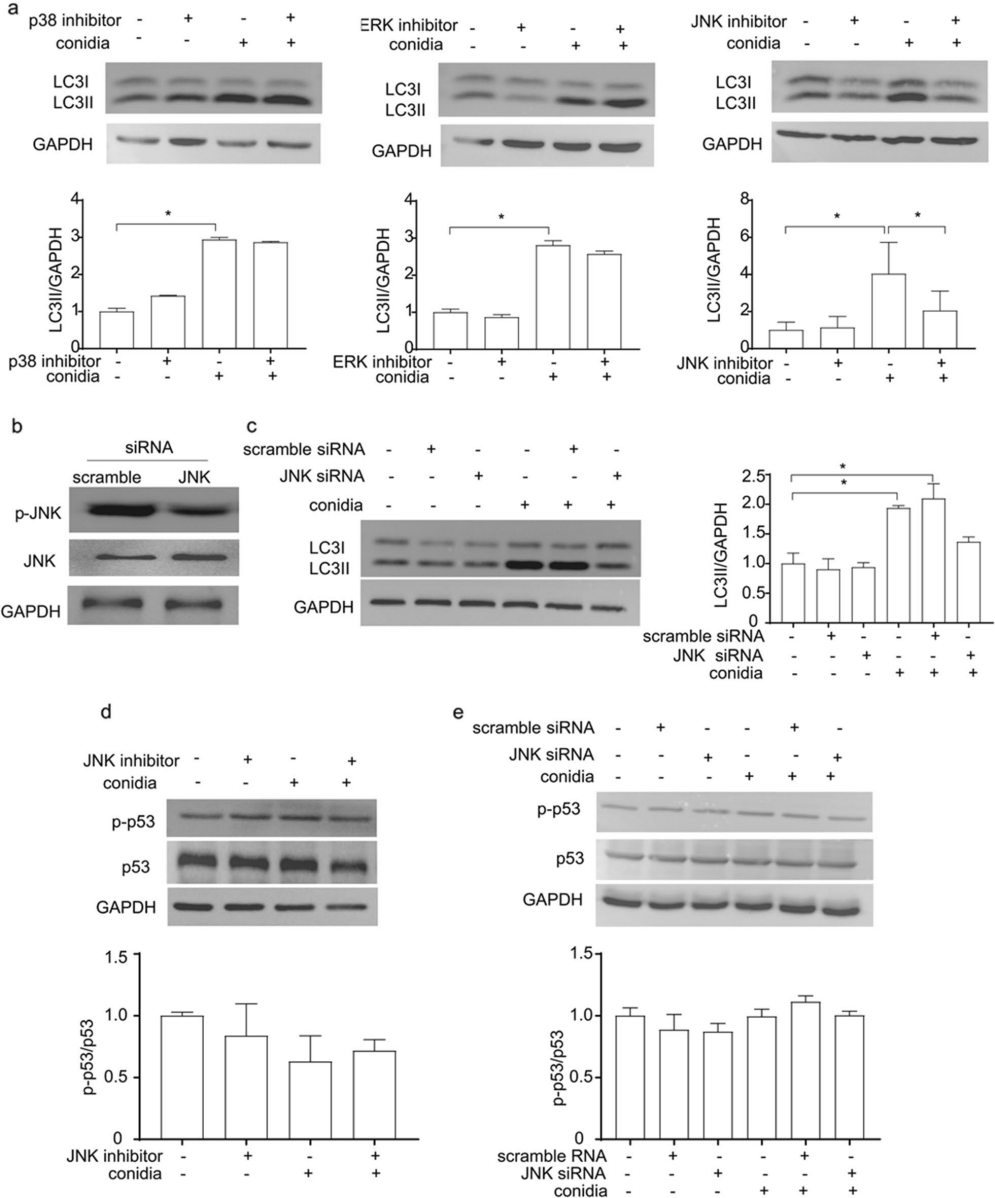
Involvement of JNK in *A. fumigatus* conidia-induced autophagy in bronchial epithelial cells. (**a**) After culturing with the corresponding inhibitor (20 μmol) for 1 h, the cells were stimulating with resting conidia for 8 h and the expression of LC3II was assessed. The blots were cut prior to hybridisation with antibodies during blotting. (**b**) Phosphorylation of JNK in BEAS-2B cells after transfected with scramble siRNA or JNK siRNA. (**c**) Expression of LC3II in BEAS-2B cells after transfected with scramble siRNA or JNK siRNA. (**d**,**e**) Prior to inoculation with *A. fumigatus* conidia for 8 h, BEAS-2B cells were treated with 20 μmol JNK inhibitor for 1 h, or transfected with siRNA targeting JNK for 36 h. Phosphorylation of p53 were measured by western blotting. The data shown as mean ± SD and represented 3 independent experiments. *P < 0.05; *n.s.* not significant.

Given that several studies have demonstrated that MAPK pathways function in the control of the balance of autophagy, we explored whether MAPK affected autophagy during *A. fumigatus* infection. As illustrated in Fig. 4a, the expression of LC3II was obviously upregulated by *A. fumigatus* conidia, which is in line with other findings in macrophages^22^. Intriguingly, increased LC3II expression was not suppressed by p38 or ERK inhibitors, but by JNK inhibitor (Fig. 4a). As a further confirmation, siRNA of JNK was used to confirm the role of JNK in *A. fumigatus-*induced autophagy. Similarly, LC3II expression was suppressed in BEAS-2B cells with significant decrease of the phosphorylation level of JNK (Fig. 4b,c). To further examine the relationship of p53 with JNK, we used inhibitor and siRNA to block JNK pathway. As shown in Fig. 4d,e, neither inhibitor nor siRNA of JNK could affect phosphorylation level of p53. These results suggested that JNK was essential for *A. fumigatus*-induced autophagy in BEAS-2B cells.

### Involvement of autophagy in the inflammatory factors release regulated by JNK signal in bronchial epithelial cells during* A. fumigatus* infection

**Figure 5:**
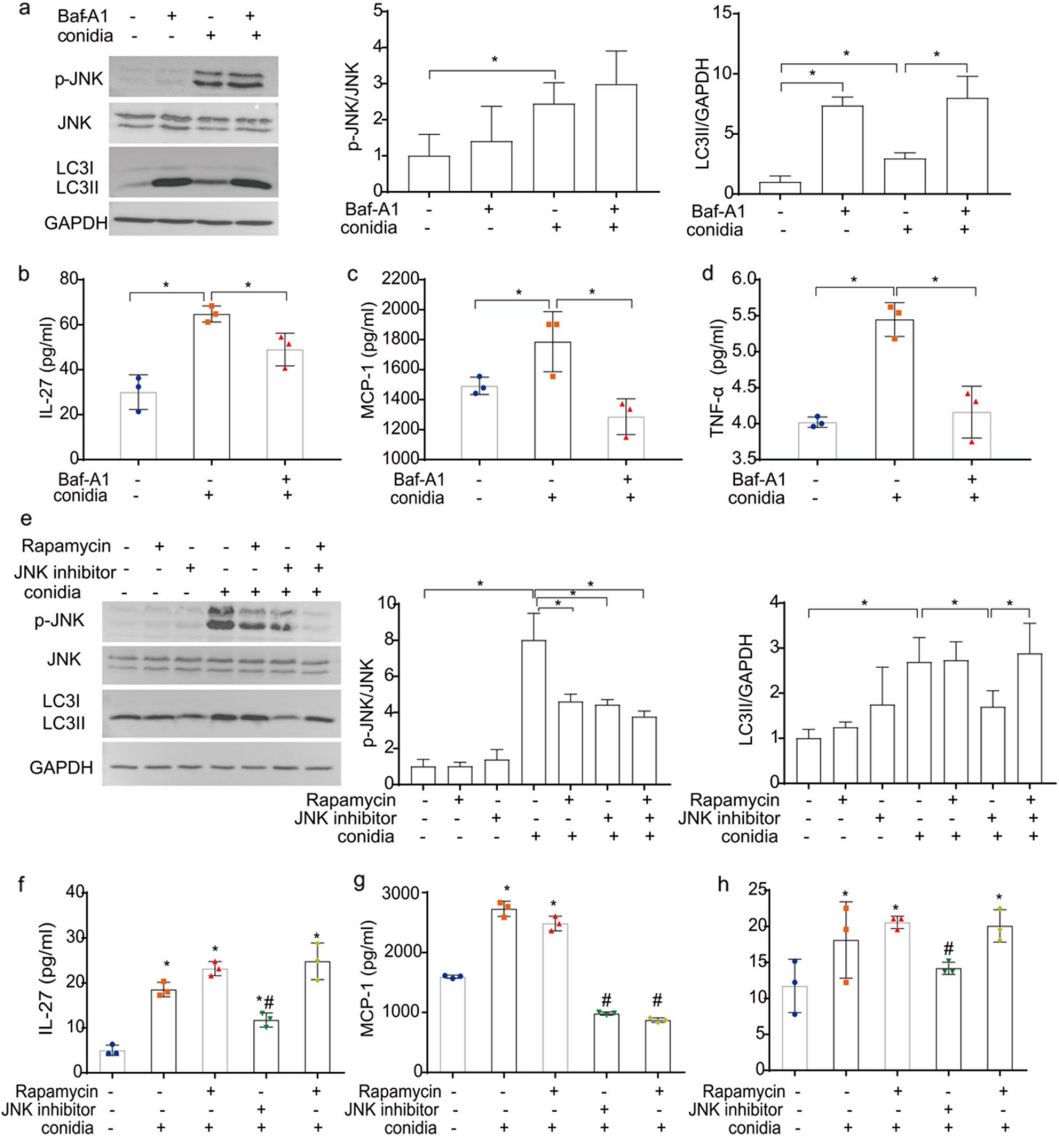
Involvement of autophagy in the release of inflammatory factors in bronchial epithelial cells by *A. fumigatus* conidia exposure. Prior to inoculation with *A. fumigatus* conidia for 8 h, BEAS-2B cells were treated with 10 nM Baf-A1 or 100 nM Rapamycin for 12 h. The expression of p-JNK, JNK and LC3II was detected by western blotting. The blots were cut prior to hybridisation with antibodies during blotting (**a**,**e**). The secretion of IL-27, MCP-1 and TNF-α in supernatant of each group was detected (**b**–**d**,**f**–**h**). Data are presented as mean ± SD (*P < 0.05 versus control group, ^#^*P* < 0.05 versus conidia stimulation group).

It was reported that autophagy could modulate the inflammation responses against pathogens infection in nonmyeloid cells^23^, it was interesting to know if the autophagy is involved in regulating the release of inflammatory factors in bronchial epithelial cells upon *A. fumigatus* stimulation. Bafilomycin A1(Baf-A1), an inhibitor of autophagosome fusion with lysosomes that leads to intracellular aggregation of LC3II, was used to pretreat BEAS-2B cells. Western blotting results showed that Baf-A1 inhibited autophagy but not conidia-induced JNK phosphorylation (Fig. 5a). Then the release of cytokines was measured. The pretreatment of Baf-A1 significantly inhibited the upregulation of IL-27, MCP-1 and TNF-α in BEAS-2B cells induced by *A. fumigatus* (*P* < 0.05) (Fig. 5b–d). These results suggested that autophagy might play an important role in regulating the release of inflammatory factors triggered by *A. fumigatus*.

Furthermore, the autophagy agonist rapamycin and JNK inhibitor SP600125 were applied to evaluate the interplay between JNK, autophagy and inflammatory factors secretion. The results showed that both rapamycin and JNK inhibitor inhibited conidia stimulated JNK phosphorylation. Moreover, administration of rapamycin reversed the inhibitory effect of JNK inhibitor on LC3II expression (Fig. 5e). We also tested the effect of rapamycin on inflammatory factors release. The result showed the administration of rapamycin reversed the inhibition of JNK inhibitor on IL-27 and TNF-α secretion, but not MCP-1 secretion (Fig. 5f–h), which suggested JNK played a role in the secretion of inflammatory factors by regulating autophagy.

## Discussion

The clearance of *A. fumigatus* conidia from the lungs relies on immune cells, including macrophages, neutrophils and airway epithelial cells^24,25^. *A. fumigatus* conidia can be internalized into immune cells, then infected immune cells respond offensively by secreting inflammatory factors^26^. Lung macrophages inhibited growth of *A. fumigatus* conidia by secreting inflammatory cytokines and fungicidal mediators^27^. Airway epithelial cells also secreted inflammatory factors after stimulation by *A. fumigatus* conidia, nevertheless, there are no convincing data about the effect of the above process. Some studies suggested that the epithelium may initiate an inflammatory response against *A. fumigatus* by releasing pro-inflammatory cytokines^26,28,29^. In the present study, we found that *A. fumigatus* alters the secretion of inflammatory factors in bronchial epithelial cells, including IL-27, MCP-1, TNF-α, MCP-3 and IP-10.

Among the cytokines, IL-27 was suggested to negatively control host immunity against *A. fumigatus*, which was evidenced by the decreased fungal colonization and less severe infection in IL-27 receptor-deficient mice^30^. MCP-1 was reported to participate in the antifungal and allergic responses to *A. fumigatus* conidia, while the elevated level of TNF-α correlates with the enhanced pulmonary inflammation and cell death^20^. Literature statements referring to change of IP-10 during *A. fumigatus* infection are contradictory. R Bals. et al.reported that respiratory epithelial cells recognized inactivated resting conidia and induced the expression of IP-10^31^, while Norton JE, et al*.* suggested that the expression of IP-10 was suppressed in BEAS-2B cells stimulated with *A. fumigatus* extract, which is consistent with our research^32^. This may be due to different cell types or conidia morphology. All these results suggested that inflammatory cytokines and airway epithelium may play a role in the pathogenesis of IPA.

The crosstalk between autophagy and inflammation in fungal infection is extensive.

Most studies reported that inflammasome is involved in the regulation of autophagy^33^. Components of inflammasomes can recruit autophagy and ward off proinflammatory cell death^34^. Meanwhile, autophagy initiated immune response and function in both the activation and inactivation of inflammation^35^. Autophagy could control inflammatory signaling by regulating inflammatory transcriptional responses and inflammasome activation^36^. As important immune response to infection, inflammation and autophagy shared many common signaling pathways, including MAPK, and so on. MAPKs include ERK, JNK and p38 MAPK, which regulate cell proliferation, differentiation, survival and innate immunity in mammals^37,38^. It has been previously reported that MAPK can be activated by many pathogens, including bacteria, fungi and viruses^39–41^. Activated MAPK can transmit extracellular signals to regulate both inflammation and autophagy. We analyzed the involvement of MAPK family in the process of *A. fumigatus* infection. The results found that *A. fumigatus* conidia could activate p38 MAPK, ERK, and JNK signaling pathways. Furthermore, JNK inhibitor reversed *A. fumigatus* induced LC3II expression and secretion of inflammatory factors in BEAS-2B cells, while the p38 MAPK and ERK inhibitors had no effect on these processes.

**Figure 6:**
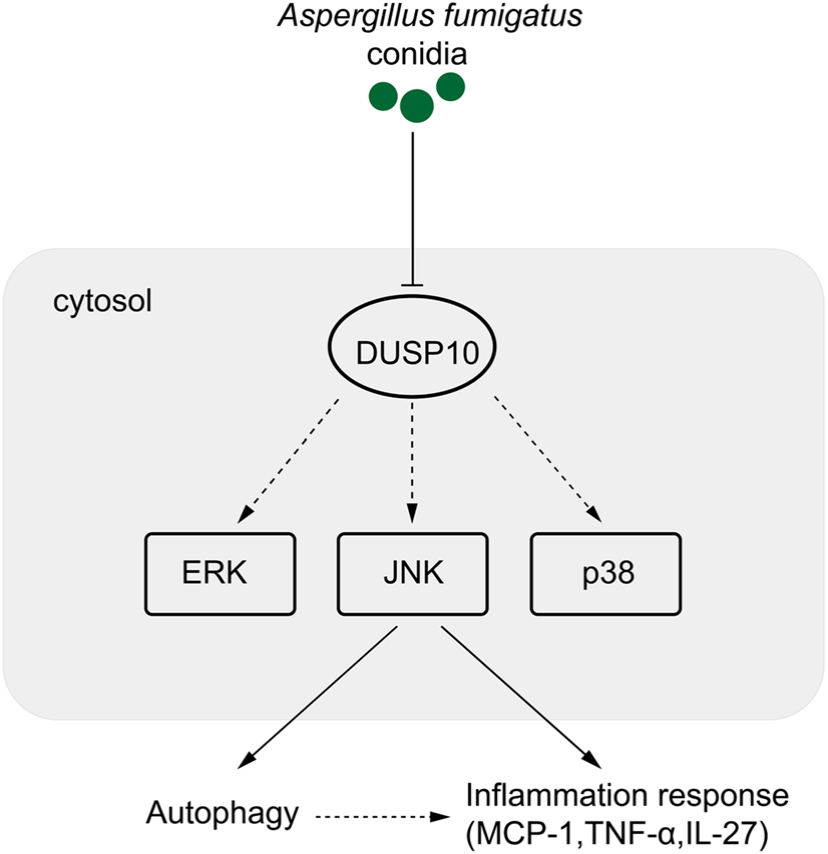
*A. fumigatus* may induce the secretion of cytokines through JNK signal relating to autophagy in bronchial epithelial cells. Molecular mechanism of inflammation and autophagy induction by *A. fumigatus* conidia. The above process was mediated by JNK pathway and independent of p38 and ERK.

Next, we studied the interaction between JNK, autophagy and inflammatory cytokines secretion. Inhibition of autophagy by Baf-A1 can regulate the secretion of inflammatory factors without affecting JNK phosphorylation, which suggested autophagy promote inflammatory activation, thus facilitating *A. fumigatus* clearance. Surprisingly, conidia-induced JNK phosphorylation was downregulated by the mTOR inhibitor rapamycin, suggesting a possible negative feedback regulation of the JNK/mTOR pathway. As the upstream signal of mTOR, MAPK/JNK activity can cause significant inhibition of the mTOR signaling pathway^37^. The inhibition of mTOR by rapamycin may negatively regulate JNK activity. Moreover, rapamycin can reverse the inhibition of inflammatory factors secretion by JNK inhibitors. Accordingly, our study has demonstrated that *A. fumigatus* might induce the secretion of cytokines through JNK signaling pathway which may relate to autophagy in bronchial epithelial cells (Fig. 6).

There are limitations in our study. Firstly, our findings were based on the experiments in the cell lines in vitro. In practice, studies on animals will be conducted to verify our results. Secondly, further investigations on the molecular mechanisms underlying how *A. fumigatus* conidia regulates JNK pathway and its downstream genes are warranted. In conclusion, our study demonstrated that *A. fumigatus* conidia induce autophagy and inflammatory factors through JNK signaling pathway in BEAS-2B cells, which might play a key role in responding to *A. fumigatus* in bronchial epithelium.

## Materials and methods

### *A. fumigatus* strain and cell line

*A. fumigatus* wild type strain B5233 was a gift from Dr. KJ.Kwon-Chung (National Institute of Health, Bethesda, Maryland)^38^. The *A. fumigatus* conidia was propagated on Sabouraud dextrose agar (10 g/L peptone, 10 g/L glucose, 15 g/L agar, pH 6.0) for 5–8 days at 37 °C and prepared as described in a previous study^39^. Briefly, after 5∼8 days culture, conidia were dislodged from agar plates with gentle washing and resuspended in sterile phosphate-buffered saline supplemented with 0.1% Tween 20. The conidia were passed through 8 layers of sterile gauze to remove hyphal fragments. Then the conidia were washed twice and enumerated on a hemacytometer. The human bronchial epithelial cell line BEAS-2B were obtained from ATCC (America Type Culture Collection) and cultured in RPMI-1640 (GIBCO, Germany) supplemented with 10% heat-inactivated foetal calf serum, 100 U/ml streptomycin, and 100 U/ml penicillin at 37 °C in an atmosphere of 5% CO_2_.

### Chemical reagents, antibodies and siRNA

P38 MAPK inhibitor SB203580, ERK inhibitor U1026, JNK inhibitor SP600125, Baf-A1 and Rapamycin were obtained from MedChemExpress (Shanghai, China**).** The above reagents were dissolved in dimethyl sulfoxide (DMSO) in the experiments. Rabbit anti-p-p53 (80195-1-RR), anti-p53 (10442-1-AP) antibody was obtained from Proteintech (Wuhan, China**)**. The other antibodies used in the experiments were purchased from Cell Signaling Technology (Maryland, USA). The HPR-conjugated goat anti-mouse IgG and HRP-conjugated goat anti-rabbit IgG antibodies were purchased from ZSGB-BIO (China). siRNAs for negative control and JNK were purchased from Ribobio (Guangzhou, China**)**.

### Treatment of BEAS-2B cells

BEAS-2B cells were added into 6-well plates (5 × 10^5^ cells/well) and cultured overnight. Medium was then changed to serum-free medium and cells were incubated with resting conidia at multiplicity of infection (MOI) = 10 for indicated time at 37 °C in 5% CO_2_. For blocking MAPK pathway, cells were incubated with 20 µM p38 MAPK inhibitor SB203580, 20 µM ERK inhibitor U1026, or 20 µM JNK inhibitor SP600125 dissolved in dimethyl sulfoxide (DMSO) for 1 h. Then, cells were incubated in serum-free 1640 medium with *A. fumigatus* resting conidia (MOI = 10) for 8 h at 37 °C in 5% CO_2_. To directly affect the autophagy process, cells were pretreatment with 10 nM Baf-A1 or 100 nM Rapamycin for 12 h. Then, cells were incubated in serum-free 1640 medium with *A. fumigatus* resting conidia (MOI = 10) for 8 h at 37 °C in 5% CO_2_. The uninfected BEAS-2B cells were cultured under the same medium conditions.

### Western blotting analysis

Cells were collected using RIPA lysis buffer with protease inhibitors, and total protein concentrations were quantified with the Pierce™ BCA Protein Assay Kit (Biomed, Beijing, China). Equivalent amounts of protein were separated on SDS-PAGE and electro-transferred to polyvinylidene difluoride (PVDF) membranes (Millipore, Germany). The blots were cut prior to hybridisation with antibodies during blotting. The membranes were blocked with 5% Bovine Serum Albumin (BSA) for 2 h and incubated with the corresponding primary antibodies at 4 °C overnight, including LC3II (#2775), p62 (#23214), p-ERK (#9101), ERK (#9102), p-JNK (#9251), JNK (#9252), p-p38 MAPK (#9211), p38 MAPK (#9218), p-p53 (80195-1-RR), p53 (10442-1-AP) and GAPDH (#5174). Then PVDF membranes were incubated with appropriate secondary antibody. Signals were detected using ECL reagent (Pierce ECL Western Blotting Substrate, Thermo Scientific).

### Analysis of *A. fumigatus* internalization

Nystatin protection method was used to investigate the internalization of *A. fumigatus* into BEAS-2B cells^40^. Cells were seeded in 96-well plates at a density of 1 × 10^4^ cells/well prior to the experiment, and pretreated with different inhibitors at 37 °C for 1 h. Then, cells were incubated with conidia of *A. fumigatus* at a multiplicity of infection (MOI) of 10 for 1 h at 37 °C in 5% CO_2_ with growth medium. The monolayers were washed 3 times with PBS and incubated with nystatin (25 μg/mL) in growth medium for 4 h at 37 °C. Subsequently, the cells were washed three times with PBS and lysed by incubating in 0.25% Triton X-100 diluted in PBS for 15 min. The released conidia were diluted and plated onto SDA agar (3 replicate plates/well) and incubated at 37 °C for 18 h. The colonies were counted to enumerate the total intracellular conidia. The internalization capacity is expressed as a percentage of the initial inoculum.

### Cytokine measurement

Milliplex human cytokine/chemokine/growth factor-immunology multiplex assay (HCYTA-60K, EMD Millipore, Billerica, MA) was used to quantify expression levels of cytokines in cell culture supernatant^41^. The following cytokines were measured: IL-27, MCP-1, TNF-α, IP-10, MCP-3, IL-15, IL-17A, IL-17E, IL-17F, IL-18, IL-22 and TNF-β. The quantification of targeted analytes was performed in accordance with the manufacturer’s instructions. When values were above or below the detectable range, the upper or lower detectable level for cytokines on standard analysis curves was used to avoid missing values. Units for all analytes are in pg/mL of fluid.

### Quantitative real-time PCR

Total RNA was extracted from cells using TRIzol reagent (Invitrogen), and the first-strand complementary DNA (cDNA) was synthesized using the RevertAid First Strand cDNA Synthesis Kit (Tian Gen, Beijing, China). Briefly, cDNA was synthesized from 1 μg of isolated total RNA, oligo-dT_18_, and reverse transcriptase in a final volume of 20 μl. Then, the cDNA was quantified with a SYBR Green Real-Time PCR Master Mix Kit (Tian Gen, Beijing, China). 1 µl of specific forward and reverse primers (10 µM), Taqase and 2 µl cDNA synthesis were mixed and performed with 40 thermocycles for 30 s at 94 °C, 5 s at 94 °C, and 30 s at 60 °C. The primers used in the assays were listed in Table 1. PCR quantification was conducted using the 2^−ΔΔCT^ method and normalized to GAPDH.

**Table 1:** Primers used in the study.

Name	Forward 5′–3′	Reverse 5′–3′	Efficiency value (%)
GAPDH	ATCCCATCACCATCTTCCAG	CCATCACGCCACAGTTTCCC	106
DUSP1	CAACGAGGCCATTGACTTCATAG	CAAACACCCTTCCTCCAGCA	106
DUSP2	AAAACCAGCCGCTCCGAC	CCAGGAACAGGTAGGGCAAG	109
DUSP4	CTGGTTCATGGAAGCCATAGAGT	CGCCCACGGCAGTCC	108
DUSP5	CCGCGGGTCTACTTCCTCA	GGGTTTTACATCCACGCAACA	89
DUSP6	CTGCCGGGCGTTCTACCT	CCAGCCAAGCAATGTACCAAG	107
DUSP7	GTGCTCGGCCTGCTCCT	GAAGAGCTGTCCACGTTGGTC	111
DUSP8	GCATCCTGCCTCACCTCTACC	CCATTTTGCGTCATCAGATCC	114
DUSP9	CTGCTGCAGAAGCTGCGA	CCTGGAATCTGCTGAAGCCT	95
DUSP10	GCCAGCCACTGACAGCAAC	TCCCACACTGGTGAGCTTCC	93
DUSP16	TCACTGTACTTCTGGGTAAACTGGAG	AAGGCTGAGAAATGCAGGTAGG	102

### Statistical analysis

Data are represented as means ± standard deviations (SD) of results from the 3 independent experiments. Statistical analysis was performed using GraphPad Prism software. The statistical significance between the indicated groups were determined by Student’s *t* test and one-way analysis of variance (ANOVA) followed by post hoc Tukey test. *P* < 0.05 was considered a statistically significant value.

## Supplementary Information

Supplementary Tables.

Supplementary Figures.

## Data Availability

All data generated or analyzed during this study are included in this published article and its supplementary information files.
