# Supplementary material for: Involvement of JNK signaling in Aspergillus fumigatus-induced inflammatory factors release in bronchial epithelial cells
Source: Sci Rep. 2023 Jan 23;13:1293. doi: 10.1038/s41598-023-28567-3 (PMC9871034; doi:10.1038/s41598-023-28567-3)

Involvement of JNK signaling in *Aspergillus fumigatus*-induced inflammatory factors release in bronchial epithelial cells

Xiao Cui[1#], Fangyan Chen[2#], Jingya Zhao[2], Dingchen Li[2], Mandong Hu[3], Xue Chen[1], Yulin Zhang[1*], Li Han[2*]

[1] Department of Respiratory and Critical Care Medicine, Beijing Youan Hospital, Capital Medical University, Beijing, 100069, China.

[2] Department for Disinfection and Infection Control, Chinese PLA Center for Disease Control and Prevention, Beijing, China.

[3] National Center of Biomedical Analysis, 27 Taiping Lu, Beijing 100850, PR China.

[#] Xiao Cui and Fangyan Chen contributed equally to this work.

[*] **Co-correspondence**

Yulin Zhang and Li Han

Department of Respiratory and Critical Care Medicine, Beijing Youan Hospital, Capital Medical University, Beijing, 100069, China.

Department for Disinfection and Infection Control, Chinese PLA Center for Disease Control and Prevention, Beijing, China.

Tel: (86) 10-83997143, Fax: (86) 10-63293371

Email: Yulin Zhang: yulinzhang@ccmu.edu.cn.

Li Han: hanlicdc@163.com

Supplementary Figure S1. The original Western blot images of Fig.2

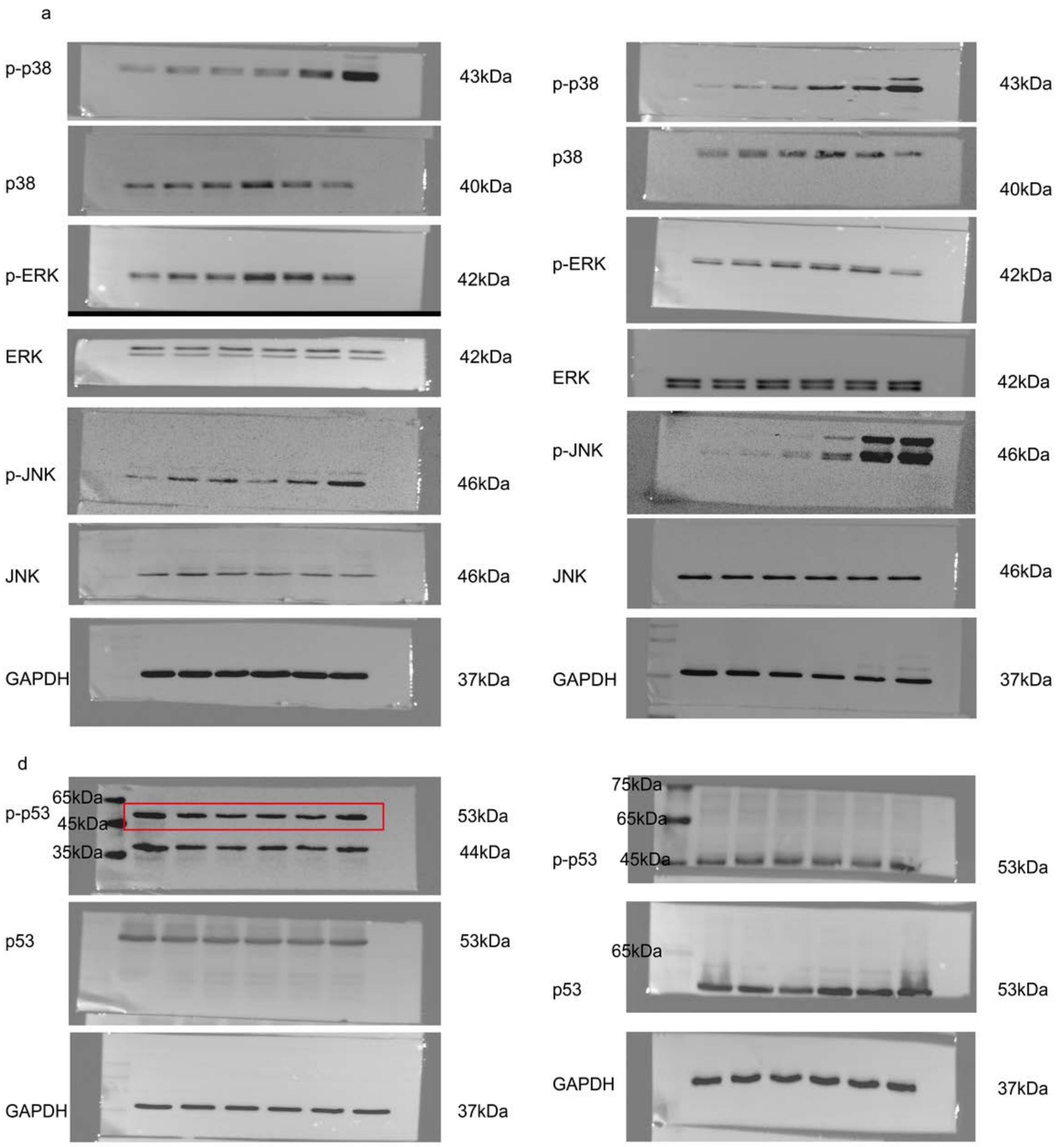

Supplementary Figure S2. The original Western blot images of Fig.4

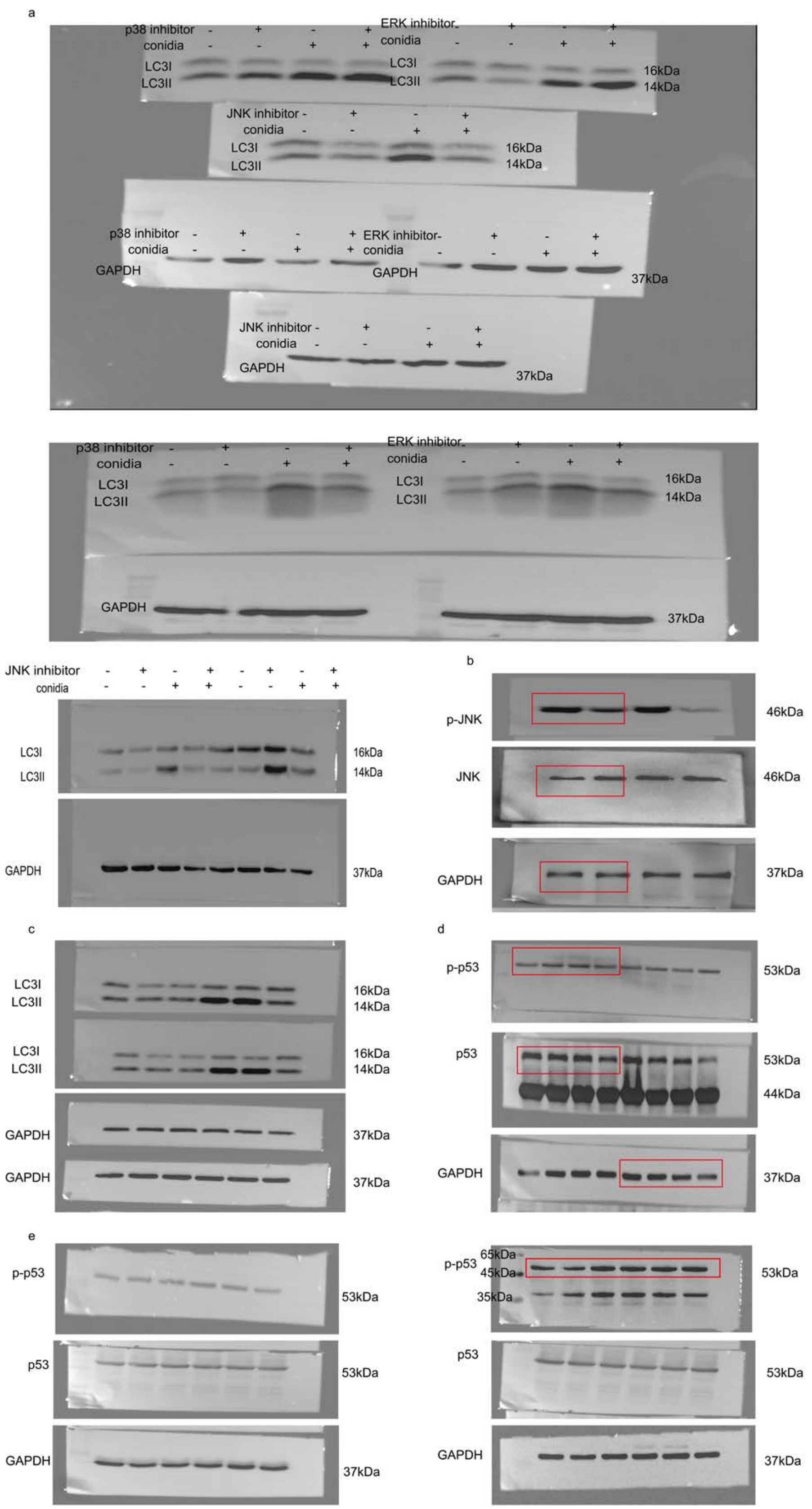

Supplementary Figure S3. The original Western blot images of Fig.5

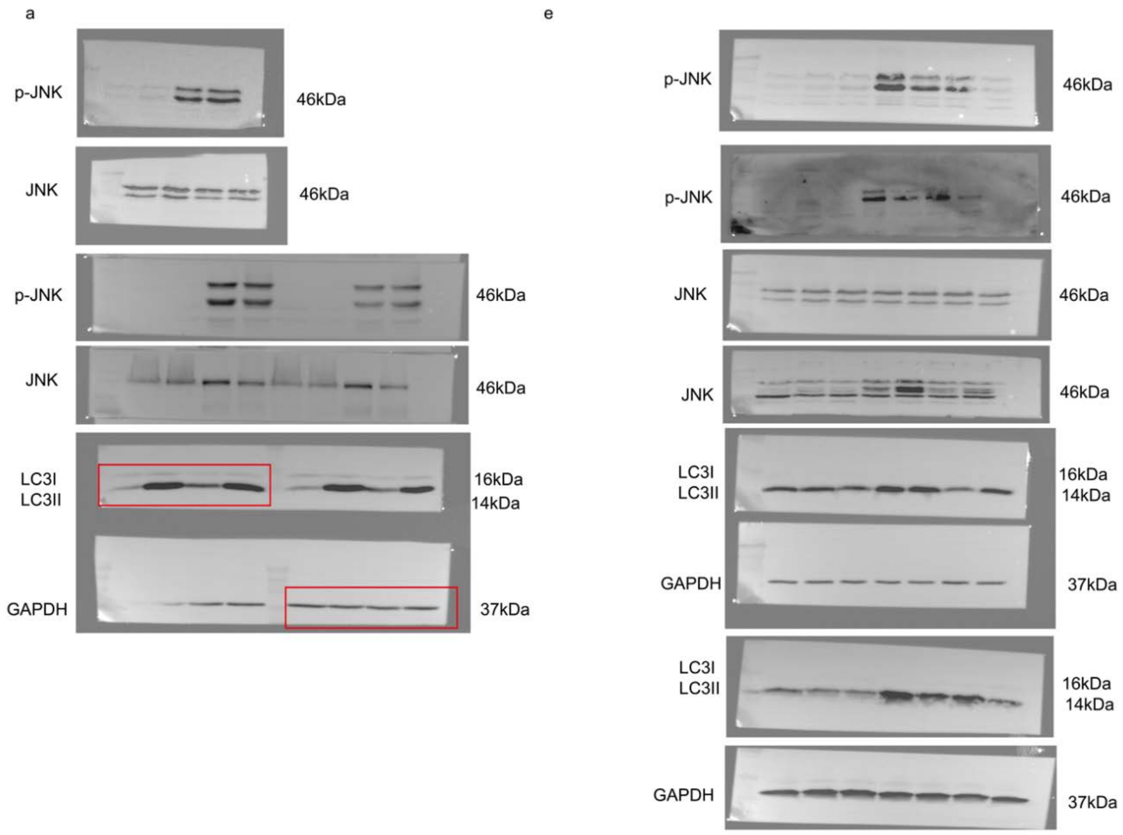

Supplement: Supplementary file 2 — Supplementary Figures. [file 41598_2023_28567_MOESM2_ESM.pdf]
